# Brain Histamine Modulates the Antidepressant-Like Effect of the 3-Iodothyroacetic Acid (TA1)

**DOI:** 10.3389/fncel.2019.00176

**Published:** 2019-05-08

**Authors:** Annunziatina Laurino, Elisa Landucci, Lorenzo Cinci, Manuela Gencarelli, Gaetano De Siena, Lorenza Bellusci, Grazia Chiellini, Laura Raimondi

**Affiliations:** ^1^Departments of Neurology, Psychology, Drug Sciences and Child Health, Section of Pharmacology, University of Florence, Florence, Italy; ^2^Department of Health Sciences, Section of Pharmacology, University of Florence, Florence, Italy; ^3^Department of Pathology, University of Pisa, Pisa, Italy

**Keywords:** 3-iodothyroacetic acid, thyroid hormone, histamine, mast cells, antidepressant effect

## Abstract

3-iodothyroacetic acid (TA1), an end metabolite of thyroid hormone, has been shown to produce behavioral effects in mice that are dependent on brain histamine. We now aim to verify whether pharmacologically administered TA1 has brain bioavailability and is able to induce histamine-dependent antidepressant-like behaviors. TA1 brain, liver and plasma levels were measured by LC/MS-MS in male CD1 mice, sacrificed 15 min after receiving a high TA1 dose (330 μgkg^–1^). The hypothalamic mTOR/AKT/GSK-β cascade activation was evaluated in mice treated with 0.4, 1.32, 4 μgkg^–1^ TA1 by Western-blot. Mast cells were visualized by immuno-histochemistry in brain slices obtained from mice treated with 4 μgkg^–1^ TA1. Histamine release triggered by TA1 (20–1000 nM) was also evaluated in mouse peritoneal mast cells. After receiving TA1 (1.32, 4 or 11 μgkg^–1^; i.p.) CD1 male mice were subjected to the forced swim (FST) and the tail suspension tests (TST). Spontaneous locomotor and exploratory activities, motor incoordination, and anxiolytic or anxiogenic effects, were evaluated. Parallel behavioral tests were also carried out in mice that, prior to receiving TA1, were pre-treated with pyrilamine (10 mgkg^–1;^ PYR) or zolantidine (5 mgkg^–1^; ZOL), histamine type 1 and type 2 receptor antagonists, respectively, or with *p*-chloro-phenylalanine (100 mgkg^–1^; PCPA), an inhibitor of serotonin synthesis. TA1 given i.p. to mice rapidly distributes in the brain, activates the hypothalamic mTOR/AKT and GSK-3β cascade and triggers mast cells degranulation. Furthermore, TA1 induces antidepressant effects and stimulates locomotion with a mechanism that appears to depend on the histaminergic system. TA1 antidepressant effect depends on brain histamine, thus highlighting a relationship between the immune system, brain inflammation and the thyroid.

## Introduction

Recent evidence indicates that thyroid hormone metabolism may generate compounds endowed of behavioral and metabolic effects.

The TA1 is the last iodinated thyroacetic acid produced by sequential metabolism of thyroid hormone carried out by deiodinases and amine oxidases activities. While little is still known about the TA1 tissue levels and their physiological significance, the pharmacological effects of TA1 have been extensively studied. In this respect, we have described that the systemic administration of low TA1 doses (μgkg^–1^) induced central and peripheral effects. The central effects that were reported include the activation of neuronal signaling, such as the pro-survival PI3K/AKT cascade (Laurino et al., 2018b), the stimulation of memory and reversion of scopolamine-induced amnesia (Musilli et al., 2014; Laurino et al., 2015a), the stimulation of wakefulness in mice prone to sleep for a high acute dose of ethanol (Laurino et al., 2018a). In addition, recent data indicate that TA1 may behave as a potent anticonvulsant and neuroprotective agent against excitotoxicity (Laurino et al., 2018b). Among peripheral effects, TA1 has been reported to induce itching, along with a reduction of noxious and painful sensitivity (Laurino et al., 2015b). Common determinants of TA1 central and peripheral effects are their rapid onset (within 15 min of administration), the fact that they were described by typical inverted U-shaped dose-effect curves, and their dependence on the activation of the histaminergic system. Such behavioral effects indicate that TA1 may cross the BBB and that histamine might derive from a site where it is “ready to be released.” However, direct evidence of brain distribution, as well as the source of histamine involvement in TA1 behavioral effects remain elusive.

In the brain, histamine is present in the histaminergic neurons, that are localized in the hypothalamus from where they project to most of the brain areas, but also in non-neuronal cells including mast cells, that are multifunctional bone marrow-derived tissue-dwelling cells and are considered one of the major sources of histamine in body tissues, including the brain. Mast cells are mainly localized along the blood vessels on the brain side of the BBB (Skaper and Fusco, 2014) and are considered crucial mediators of glial cells-neurons communication, whose main signaling pathway is represented by the activation of the PI3K/AKT cascade (Dong et al., 2017). At the BBB, mast cells represent the first-line of defense against brain invasion by xenobiotics, quickly releasing pre-stored and newly synthesized mediators, including histamine, serotonin and other pro-inflammatory signals (Theoharides and Cochrane, 2004). Despite the low number of mast cells in the healthy brain, it is estimated they can store up to 50% of the brain histamine (Yamatodani et al., 1982). Potentially, mast cells may be a target for TA1. Indeed, recent evidence indicates that histamine, irrespective of its derivation (neuronal and not neuronal), is implicated in the regulation of feeding and sleep-awake cycle (Lin et al., 1986), as well as in the stimulation of locomotion and emotional behaviors, including modulation of anxiety and depression (Lamberti et al., 1998; Wada et al., 1991; Chikahisa et al., 2013).

The aim of the present work is to investigate whether TA1: (i) has brain bioavailability after systemic administration, (ii) can trigger histamine release from mast cells and (iii) is endowed of histamine-dependent antidepressant-like effects in rodents.

## Materials and Methods

### Animals

Male CD1 mice (weight: 20–30 g) purchased from ENVIGO (Italy) were used in the present study. Five mice were housed per cage. Cages were placed in the experimental room 24 h prior to testing to ensure adaptation. Animals were housed at 23 ± 1°C under a 12 h light–dark cycle (lights on at 07:00) and were fed a standard laboratory diet with *ad libitum* access to water. Experiments and animal use procedures were in accordance with the National Institutes of Health Guide for the Care and Use of Laboratory Animals (NIH Publications No. 80–23, revised 1996). The experimental protocols were approved by the ethical Committee of the Italian Council of Health, in compliance with the European Convention for the Protection of Vertebrate Animals used for Experimental and Other Scientific Purposes (ETS no. 123) and the European Communities Council Directive of 24 November 1986 (86/609/EEC). The authors further attest that all efforts were made to minimize the number of animals used and their suffering.

For this study we received the permission from the Ethical Committee for animal health 176/2017-PR) from the Italian Ministry of Health.

Male CD1 mice (20–30 g) from ENVIGO (Italy) were used. The animals were kept at 22 ± 1°C with a 12 h light–dark cycle (light on at 07:00 h) and were fed a standard laboratory diet with water *ad libitum*. Five mice were housed per cage.

### Determination of TA1 Brain, Liver and Plasma Distribution After Systemic Administration

Twenty male mice were divided in two groups of 10 mice each. One group received injection i.p. of a saline solution (control mice), while the other received 330 μgkg^–1^ TA1 dissolved in saline solution (Veh) (Laurino et al., 2018a). This dose which was approximately ten times higher than the doses of TA1 found active on mice behavior, Was chosen to be sure to detect TA1 levels in the analyzed tissues.

Five mice from each group were sacrificed 15 min after TA1 administration, the remaining mice (five from each group) were sacrificed after 60 min. At each time point, the blood, the brain and the liver were removed and quickly frozen until used to assess the TA levels by liquid chromatography tandem mass spectrometry (LC/MS-MS). Due to the small amount of plasma obtained, TA1 determination was carried on a pooled plasma sample from five control or treated mice (at 15 and 60 min after treatment).

Assays were performed on plasma (300 μL), brain and liver samples (130–190 mg). Each sample was placed in a 2 mL Precellys tube, 1 ml of 85:15 (v/v) Acetonitrile/0.1 M HCl _(aq)_ solution containing 5 nM internal standard (TA1-D4) was added, and the sample was sonicated for 30 minutes and then homogenized using a Precellys 24 beads grinder (2.8 mm ceramic, zirconium oxide, beads). The homogenate of each sample was placed in an ultrasound bath (LBS1 3Lt, Falc Instruments, Treviglio, Italy) for 15 minutes and then centrifuged for 15 min at 1300 × *g* at room temperature. The residual pellet was discarded and the supernatant was placed in a new 12 ml glass centrifuge tube. The solution was subjected to liquid/liquid extraction with hexane (3 mL ×1 mL). The upper phase (hexane) was discarded and the lower phase (acetonitrile) was dried under a gentle stream of nitrogen at 45°C. Samples were then dissolved in 100 μL reconstitution solvent mixture (H_2_O:MeOH, 70:30) and analyzed using LC-MS/MS to assess TA1 concentration as described elsewhere (Saba et al., 2010).

### *In vivo* Mast Cells Staining

CD1 mice were treated intraperitoneally with Veh or 4 μgkg^–1^ TA1. After 15 min from injection, animals were sacrificed by CO_2_ inhalation and brains were collected and fixed for 24 h in Mota fluid (1% lead acetate in 49.57% Absolute ethanol 49.75% Water and 0.5% Acetic acid), dehydrated in graded ethanol and embedded in paraffin. The presence of mast cells and their content in secretion granules were highlighted by both conventional histological staining and histochemistry. In particular, 5 μm thick histological coronal sections collected at the hippocampus level, were stained with Astra blue (Fluka, Buchs, Switzerland). This cationic dye binds specifically to heparin contained in the mast cell granules (Cinci et al., 2010). Mast cells were histochemically labeled with FITC conjugated avidin (1:400; Sigma Aldrich, Milan, Italy). Avidin is able to electrostatically bind with high sensitivity to mast cell granules (Bacci et al., 2014).

### Mice Peritoneal Mast Cells Isolation and Culture: The Effect of TA1 on Histamine Release

Mast cells were isolated from peritoneum of CD1 mice as described in Meurer et al. (Meurer et al., 2016). Briefly, a small incision below the sternum of the animal was performed without puncturing the peritoneum. 10 ml of ice cold sterile PBS were injected in the peritoneal cavity and a soft massage of about 30 s was performed. Cell suspension was centrifuged at 4°C at 300 × *g* for 10 min, re-suspended in 5 ml of PBS and then centrifuged again at 4°C at 300 × *g* for 10 min. Cells were suspended in RPMI medium and incubated at 37°C and 5% CO_2_. After 3 days, not adherent cells were removed and fresh culture medium was added. After 6 days, 5 ml of fresh medium were added and at day 10 mast cells (represented by the non-adherent population) were harvested and used for experiments.

Mast cells were plated in 12 well plate (about 25000 cells/well) and treated with vehicle (PBS) alone, 5 mg/ml *Apis mellifera* venom (ENTOMON s.a.s Florence, Italy, gently gifted by Prof. Stefano Turillazzi and prepared in vehicle) or 20 nM, 100 nM, and 1 μM of TA1. Culture media were harvested after 5, 15, 30, 60, 90, and 120 min after treatment. Histamine was measured by the method described in Tiligada et al. (2000) with some modifications. Briefly, 50 μl of each sample was incubated with 12.5 μl of 0.44 M of NaOH+ 0.1% O-phthaldialdehyde (Sigma Aldrich, Milan, Italy) for 10 min at RT. Then the reaction was stopped by adding 12.5 μl of 0.5 M HCl and fluorescence was read by a microplate reader (360 nm excitation and 450 nm emission). Results for each treatment were expressed as baseline-corrected (Veh) fluorescence.

Vehicle or 1 μM TA1 treated cells were placed on a slide after 15 min of treatment and stained with Astra blue to visualize histamine content.

### Determination of Signaling Activity of TA1 at Hypothalamus

Twelve male CD1 mice were randomly divided in 4 groups of 3 mice each. One group received i.p. Veh the other mice received 0.4 or 1.32 or 4 μgkg^–1^ TA1. Mice were sacrificed 15 min after administration to remove the hypothalamus. The tissue was frozen at −80°C until used for western-blot analysis.

### Western Blot

Proteins (20 μg) isolated from mouse hypothalamus were separated via 4–20% SDS-PAGE and transferred into PVDF membranes (60 min at 398 mA) using standard procedures. Blots were incubated overnight at 4°C with specific antibodies against p- AKT S473, AKT, p-GSK-3β S9, GSK-3β, p-mTOR S2448 and mTOR (Cell Signaling Technology, Denver, MA, United States) and GAPDH (Merk-Millipore, Darmstadt, Germany). Primary antibodies were diluted in PBS containing 1% albumin or 5% non-fat dry milk and 0.05% Tween. The antigen–antibody complexes were visualized using appropriate secondary antibodies (1:10 000, diluted in PBS containing 1% albumin or 5% non-fat dry milk and 0.05% Tween) and incubated for 1 h at room temperature. Blots were then extensively washed with PBS containing 0.1% Tween and developed using an enhanced chemiluminescence detection system (Pierce, Rodano, Italy). Exposition and developing time were standardized for all blots. Densitometric analysis of scanned images was performed on a Macintosh iMac computer using the public domain NIH Image J program. Results are presented as the mean ± SEM of different gels and expressed as arbitrary units (AU), which depict the ratio between levels of target phosphorylated protein and the total protein expression normalized to basal levels.

## Behavioral Studies

### Treatments

Mice were randomized to receive i.p., administration of saline solution (Veh), or 1.32 or 4 μgkg^–1^ TA1 dissolved in Veh (Laurino et al., 2018a). Experiments were also performed in mice pre-treated i.p. with Veh or with antagonists of type 1 and type 2 histamine receptors, i.e., pyrilamine (10 mgkg^–1^; PYR, prepared in Veh) and zolantidine (5 mgkg^–1^ i.p.; ZOL, prepared in Veh), 20 min before they received 4 μgkg^–1^ TA1 or Veh. When p**-**chloro-phenylalanine (100 mgkg^–^1; PCPA), the inhibitor of the tryptophan hydroxylase activity was used 4 μgkg^–1^ TA1 or Veh, were administered to mice which had received a daily injection of PCPA for 4 days. TA1 or Veh were administered 1 h after the last PCPA injection. All drugs were administered at a volume of 10 mlkg^–1^ body weight.

Mouse behavior was observed 15 min after TA1 administration according to the specific methods listed below.

### The Hole Board-Platform

The hole-board test was performed according to (Romanelli et al., 2006). The experimental setting consisted of a 40 cm square plane with 16 flush mounted cylindrical holes (3 cm diameter), distributed four by four in an equidistant, grid-like manner. Mice were placed in the center of the board one by one and allowed to move about freely for a period of 5 min. Two electric eyes, crossing the plane from midpoint to midpoint of the opposite sides, thus dividing the plane into four equal quadrants, automatically signaled mouse movements (locomotor activity). Miniature photoelectric cells in each of the 16 holes recorded hole exploration. Animal groups consisted of 10 mice and were tested 10 min after the injections.

### The Rota-Road Test

The integrity of the animals’ motor coordination was assessed using a rota-rod apparatus at a rotating speed of 24 rpm. The numbers of falls from the rod in 30 s, 15 min after drug administration were counted.

### The Forced Swimming Test (FST)

The test was conducted as described by Porsolt et al. (1977). Mice (10 animals/group) were individually forced to swim in an open cylindrical container (diameter 10 cm, height 25 cm), containing 19 cm of water at 25 ± 1°C. In the test, the time of immobility was measured during a 6-min period. A decrease in the duration of immobility is indicative of an antidepressant effect.

### The Tail Suspension Test (TST)

A piece of tape was adhered to the upper middle of the tail of each animal, creating a flap with the overlap of tape. Mice were suspended from a plastic rod mounted 50 cm above the surface by fastening the tail to the rod with adhesive tape. The duration of the test was 6 min. Immobility was defined as the absence of any limb or body movements, except those caused by respiration.

### The Light Dark Box

The light–dark box was made of white and black opaque apparatus (length 50 cm, width 20.5 cm, and height 19 cm) consisted of two equal acrylic compartments, one dark and one white, illuminated by a 60-W bulb lamp and separated by a divider with a 10 cm × 3.2 cm opening at floor level. Each mouse was placed in the middle of the light chamber facing a side away from the door and then released. Mice’ behaviors were scored for 300 s and included the latency to the first step into the dark compartment, the duration of time spent in the light chamber, the number of full-body transitions between chambers. These behaviors have previously been measured as a reflection of anxiety in this apparatus (Bourin and Hascoët, 2003). After testing, subjects were removed from the light–dark box and returned to their home cage in colony room. The apparatus was cleaned with 70% ethanol after each use and allowed to dry before the next subject was tested.

### Statistical Analysis

Data are expressed as mean ± SEM of independent experiments. Statistical analysis was performed by the One or Two way ANOVA test followed by Tukey or Dunnett or Bonferroni Multiple Comparison Test. The threshold of statistical significance was set at *P* < 0.05. Data analysis was performed using the GraphPad Prism 5.0 statistical program (GraphPad software, San Diego, CA, United States).

## Results

### Endogenous and Pharmacological Tissue Levels of TA1

3-iodothyroacetic acid endogenous or pharmacological tissue levels were measured by liquid chromatography tandem mass spectrometry (LC-MS/MS) in mice sacrificed 15 min after treatment with Veh or with 330 μgkg^–1^ TA1 (Chiellini et al., 2012).

In Veh-treated mice, we were unable to measure TA1 endogenous levels in the brain and in the pooled plasma sample, whereas TA1 was detected in liver (Table 1).

**TABLE 1:** 3-iodothyroacetic acid (TA1) levels in mice following intra-peritoneal administration (i.p.) of TA1 (330 μgkg^–1^).

Treatments	Plasma	Brain (ng/g of tissues)	Liver
Saline	N.D.	N.D.	0.64±0.2
TA1 (15 min)	63.7^a^	4.2 ± 1	106±22
	19%°°	1.3%	32%
TA1 (60 min)	9.4^a^	1.1 ± 0.3	10.47±0.45
	2.8%	0.33%	3.17%

*^a^from pooled blood samples. °°The percentage of TA1 recovered in respect of the administered dose (330 μgkg^–1^).*

In TA1-treated mice, 15 min after pharmacological administration, TA1 was recovered in the pooled plasma sample (19% of the dose administered), in the brain and in the liver of all the mice treated (1.3 and 32% of the dose administered, respectively). In the liver, pharmacological administration produced a 165-fold increase of TA1 level (ng/g) when compared to endogenous levels.

As expected, TA1 tissue levels reduced with time. After 60 min from TA1 administration, plasma and liver levels decreased in parallel resulting approximately ten times lower than those measured at 15 min. In the brain, TA1 levels at 60 min were only four times lower than those measured at 15 min. These results suggest that tissues have different TA1 clearance capacity, with the brain conserving TA1 levels for longer than plasma or liver (Table 1).

### TA1 Degranulates Brain Mast Cells

We then investigated whether treatment with 4 μgkg^–1^ TA1 was associated with brain mast cells degranulation.

**FIGURE 1:**
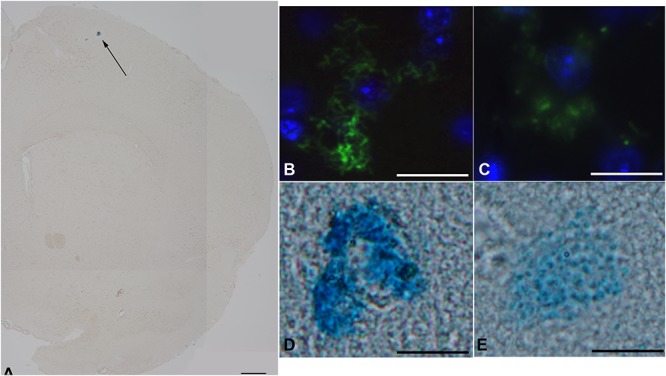
The effect of TA1 on brain mast cell degranulation. Mast cells were stained as described in Methods. **(A)** Astra blue staining, representative image of a hemisphere. Few mast cells are visible in the upper portion (black arrows) 35x magnification; scale bar 600 μm. **(B,C)** Representative images of mast cells exposed to Veh (picture B) or to 4 μgkg^–1^ TA1 **(C)** stained with FITC conjugated avidin (green). **(D,E)** Representative images of Astra blue stained mast cells exposed to Veh **(D)** or to 4 μgkg^–1^ TA1 **(E)**. 1000× magnification; scale bar 100 μm.

Our results indicated that, at our staining conditions, i.e., both avidin and Astra Blue, very few mast cells were detectable in the brain. Although the low number of mast cells could be considered as a sign of the extreme specificity of our staining protocols, we could also not exclude that the method of tissue fixation may not allow the retention of mast cells granular content in the central portion of the brain (Figure 1A). These technical considerations/limitations notwithstanding, we were still able to highlight differences in mast cell granular content between the two experimental groups (Figures 1B–E).

In fact, in Veh-treated mice, the mast cells detected showed substantially intact granular content (Figures 1B,D). Instead, in brain slices prepared from mice treated with 4 μgkg^–1^ TA1, the few mast cells that were stained showed reduced granular content (Figures 1C,E).

### TA1 Releases Histamine From Peritoneal Mast Cells

To confirm the capacity of TA1 to trigger histamine release from mast cells, we performed *in vitro* experiments exposing isolated mouse peritoneal mast cells to increasing concentrations of TA1.

**FIGURE 2:**
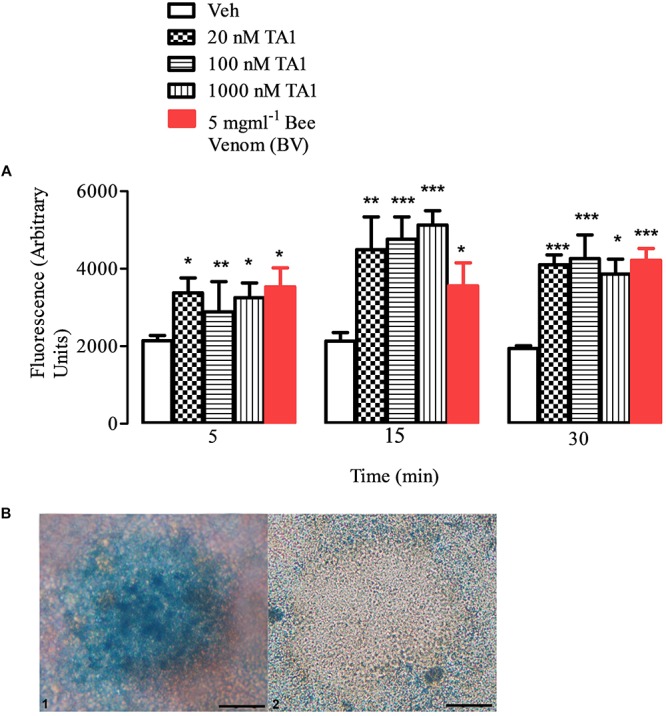
TA1 releases histamine from peritoneal mast cells. Peritoneal mast cells were prepared and exposed to 20, 100, or 1000 nM TA1 as described in Methods. **(A)** Histamine accumulation was evaluated by fluorescence in cell medium after 5, 15, 30 min of cell exposure to TA1 or to *Apis mellifera* venom (BV; 5 mgml^–1^). Results are expressed as means ± SEM of three different experiments run in duplicate (*P < 0.05, **P < 0.01 and ***P < 0.001 vs. Veh.). **(B)** Picture 1: mast cells stained with Astra Blue following 15 min exposure to Veh. A representative image is showed. Picture 2: representative image of mast cells stained with Astra Blue following 15 min exposure to 1 μM TA1.

Our results indicated that TA1 effectively degranulated peritoneal mast cells (Figures 2A,B). In fact, histamine levels in cell medium significantly increased (compared to Veh) when mast cells were exposed to increasing TA1 concentrations (Figure 2A) or to bee venom (BV) and mast cells appear degranulated (Figure 2B).

In particular, the Two-way ANOVA test indicated that, at the concentrations tested, TA1 significantly increased histamine release at all the times of cell exposure without a clear concentration dependent effect on the time of exposure. Kinetic data indicated that the releasing capacity of the acid showed a trend to increase from 5 to 15 min of cell exposure and then it remained almost stable up to 30 min (Figure 2A) indicating granular histamine depauperation in the absence of a fast re-synthesis.

### TA1 Activates the Hypothalamic AKT/mTOR and Reduces the GSK-3β Activity

Since kinetic data indicated the presence of TA1 in the brain of mice 15 min after administration, we investigated whether TA1 was able to activate any signaling activity when administered at the pharmacological doses previously found to induce behavioral effects (Laurino et al., 2018a). For this aim, mice were sacrificed 15 min after treatment with 4 μgkg^–1^ TA1 or vehicle (Veh) and the hypothalamus was isolated.

**FIGURE 3:**
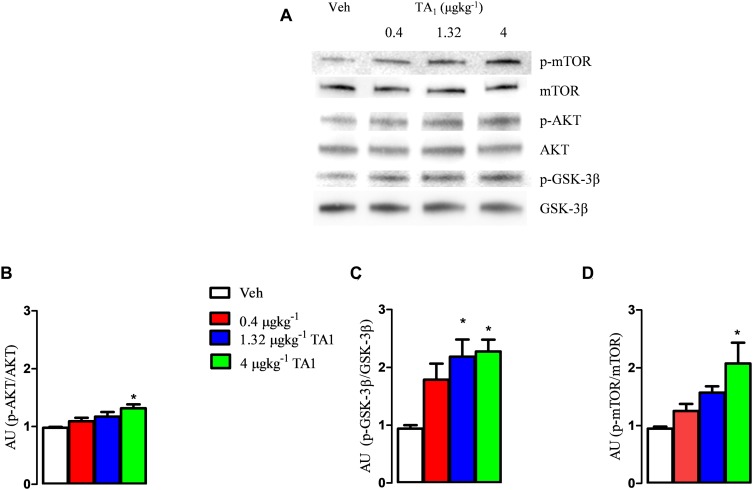
The effects of TA1 on hypothalamic p-PI3K/AKT and p GSK-3 levels. Male CD1 mice received an intraperitoneal injection of either vehicle (Veh) or 0.4, 1.32 and 4 μgkg^–1^ TA1. Hypothalamic levels of p-AKT **(B)**, p-GSK-3β **(C)**, and p-mTOR **(D)** were assessed 15 min after mice treatment. **(A)** A representative experiment is shown. **(B–D)** Densitometric analysis is showed. Results are presented as the mean ± SEM of 3 different experiments; *P < 0.05 vs. Veh. (One-way ANOVA test followed by Tukey Multiple Comparison Test).

Our results showed that the treatment with TA1 produced activation of the AKT/p-mTOR/GSK-3β cascade depending on the dose administered. In fact, while p-GSK-3β levels resulted significantly increased in the hypothalamus of mice that received 1.32 and 4 μgkg^–1^ TA1 (Figures 3A,C; *P < 0.05 vs. Veh), the levels of p-AKT and p-mTOR were found increased over Veh only in those mice treated with 4 μgkg^–1^ TA1 (Figures 3A–D, One-way ANOVA test followed by Tukey *post hoc* test; *P < 0.05 vs. Veh).

## Ta1 Treatment Induces Antidepressant-Like Effects

### TA1 Reduces the Immobility Time in the FST

The FST is a validated methodological tool for pre-clinical assessment of antidepressant drug activity. In this test, the behavioral immobility of the mice represents a condition of “despair,” since the mice give up moving after realizing that escape is impossible. Consistently, we performed the FST to investigate the possible antidepressant effects of TA1.

**FIGURE 4:**
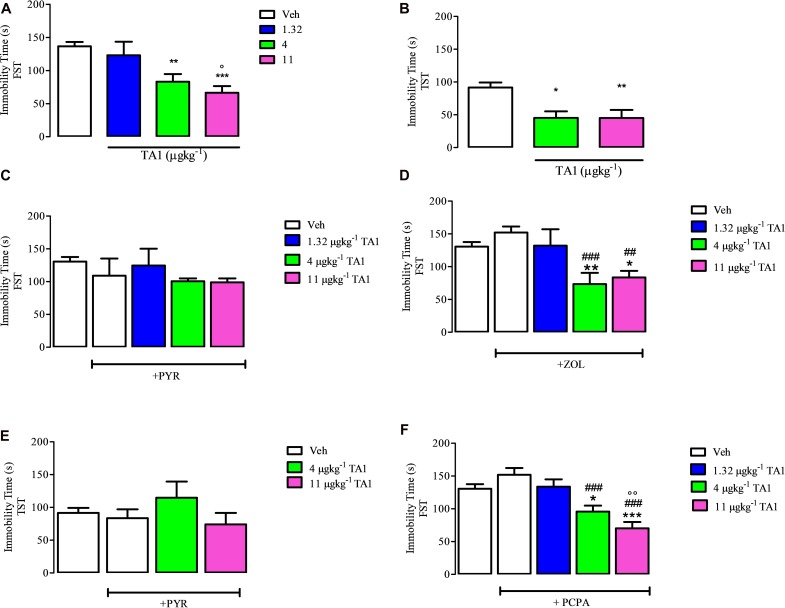
The effect of 3-iodothyroacetic acid (TA1) treatment on the immobility time in the forced swim test (FST) and in the tail suspension test (TST): evidence for the involvement of histamine and not of serotonin. Mice received 1.32, 4 and 11 μgkg^–1^ TA1 and after 15 min they were subjected to the FST **(A)** or to TST **(B)**. The immobility time was measured as described in Methods. **(A)** The immobility time measured in mice subjected to the FST is reported. Results are the means ± SEM of three different experiments were at least ten mice were used (**P < 0.01 and ***P < 0.001 vs. Veh and °*P* < 0.05 vs. 1.32 μgkg^–1^ TA1; One-way ANOVA test followed by Tukey Multiple Comparison Test). **(B)** Immobility time measured in mice subjected to the TST is reported. Results are the means ± SEM of three different experiments using at least ten mice (*P < 0.05 and **P < 0.01 vs. Veh; One-way ANOVA test followed by Tukey Multiple Comparison Test). **(C,D)** The FST was then repeated in mice pre-treated with Vehicle (Veh) or with pyrilamine (10 mgkg^–1^; PYR; **C**) or with zolantidine (5 mgkg^–1^, ZOL; **D**) before receiving 4 or 11 μgkg^–1^ TA1 as described in Methods. The Immobility time was measured. Results are the means ± SEM of three different experiments were at least ten mice were used (*P < 0.05 and **P < 0.01 vs. Veh; ###*P* < 0.001 and ##*P* < 0.01 vs. ZOL, One-way ANOVA test followed by Tukey Multiple Comparison Test); **(E)** The TST was repeated in mice pre-treated with pyrilamine (10 mgkg^–1^; PYR) before receiving 4 and 11 μgkg^–1^ TA1 as described in Methods. The Immobility time was measured. Results are the means ± SEM of three different experiments were at least ten mice were used (One-way ANOVA test followed by Tukey Multiple Comparison Test); **(F)** the FST was repeated in mice pre-treated for 4 days with p-chloro methyltyrosine (100 mgkg^–1^; PCPA) and then with 1.32, 4 and 11 μgkg^–1^ TA1 as described in Methods. The Immobility time was measured. Results are the means ± SEM of three different experiments were at least ten mice were used (*P < 0.05 and ***P < 0.001 vs. Veh, ###*P* < 0.001 vs. PCPA, °°*P* < 0.01 vs. 1.32 μgkg^–1^; One-way ANOVA test followed by Tukey Multiple Comparison Test).

One-way ANOVA analysis of the data obtained from the FST indicated that 4 and 11 μgkg^–1^ TA1 significantly reduced the immobility time (**P < 0.01 and ***P < 0.001 vs. Veh. respectively and °*P* < 0.05 vs. 1.32 μgkg^–1^ TA1 *post hoc* Tukey Multiple Comparison Test), thus supporting a possible antidepressant effect of TA1, which required to be confirmed by additional tests (Figure 4A).

### TA1 Reduces the Immobility Time in the TST

The TST is a test based on the assumption that the animal will try to escape the stressful situation (i.e., mice suspended). After a certain time, the animal ceases to struggle and immobility occurs. As for the FST, longer immobility times are sign of depressive behavior.

Mice were subjected to the TST in order to confirm the results obtained in the FST. Results showed that 4 and 11 μgkg^–1^ TA1 treatment significantly reduced the immobility time in the TST too (*P < 0.05 and **P < 0.01 vs. Veh treated mice; Figure 4B).

### The Antidepressant Effect of TA1 Includes the Activation of the Histaminergic System

As previously reported, the behavioral effects induced by TA1 resulted to be mediated by the activation of the histaminergic system, since these actions are modulated by anti-histaminergic drugs (Laurino et al., 2015a, 2017a, b). In line with this evidence, we aimed to investigate whether the antidepressant effects of TA1 were also modulated by treatment with anti-histaminergic drugs (i.e., PYR and ZOL).

As a control, the treatment of mice with ZOL and PYR alone did not affect the immobility time of mice in both FST and TST tests in respect of Vehicle (Veh) treated mice (One-way Anova test followed by Tukey Multiple Comparison test Figures 4C–E).

In mice pre-treated with 10 mgkg^–1^ PYR, TA1, at all the doses tested, failed to reduce the immobility time of the mice (Figure 4C). Instead, in mice pre-treated with 5 mgkg^–1^ ZOL, 4 and 11 μgkg^–1^ TA1 significantly reduced the immobility time in the FST (*P < 0.05 and **P < 0.01 vs. Veh alone respectively, ###*P* < 0.001 and ##*P* < 0.01 vs. ZOL respectively, Tukey Multiple Comparison Test analysis) (Figure 4D).

Consistently, in the TST, in mice pre-treated with 10 mgkg^–1^ PYR, TA1 4 and 11 μgkg^–1^ did not induce any significantly reduction of the immobility time of the mice (Figure 4E).

### The Antidepressant Effect of TA1 Does Not Include the Serotoninergic System

To investigate the involvement of serotonin in TA1 antidepressant effects, we performed the FST on mice deprived of serotonin after a 4 day treatment with PCPA, an inhibitor of the tryptophan hydroxylase activity.

Our results showed that mice treated for 4 days with 100 mgkg^–1^ PCPA had immobility time similar to Veh-treated mice (Figure 4F, One-Way Anova test followed by Tukey Multiple Comparison test). At this condition, the administration of 4 and 11 μgkg^–1^ TA1 still significantly reduced the immobility time vs. Veh-treated mice (Figure 4F; *P < 0.05 and ***P < 0.001 vs. Veh, ###*P* < 0.001 vs. PCPA, °°°*P* < 0.01 vs. 1.32 μgkg^–1^ TA1, Tukey Multiple Comparison Test). These data indicated that the serotoninergic system was not involved in TA1 antidepressant effect.

### TA1 Does Not Show Anxiolytic-Like Effects

**FIGURE 5:**
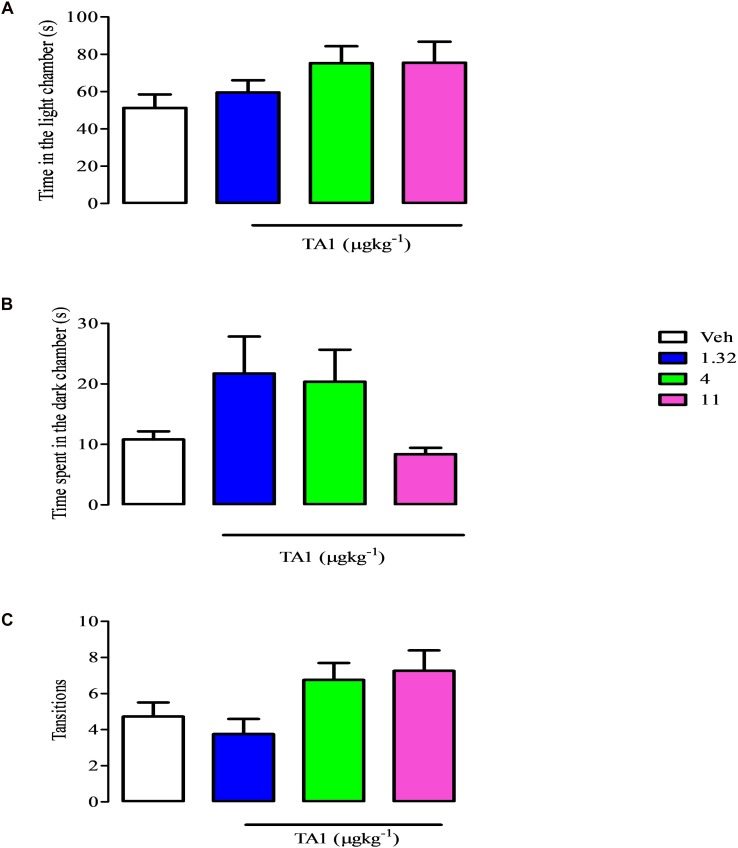
TA1 does not show anxyolitic-like effects. The light dark paradigm was carried on mice received 1.32, 4 and 11 μgkg^–1^ TA1 or vehicle (Veh) as described in Methods. Results are expressed as the means ± SEM of measurements relative to 10 mice. Mice behaviors were scored for 300 sec and included **(A)** the duration of time spent in the light chamber, **(B)** the latency to the first step into the dark compartment, **(C)** the number of full-body transitions between chambers.

The antidepressant effect of drugs might derive from their anxiolytic features. To exclude that TA1 was endowed of anxiolytic effects we performed the light-dark box test with mice which received 4 and 11 μgkg^–1^ TA1. The One-way Anova test of results followed by Dunnett’s *post hoc* test indicated that mice treated with TA1 and Veh spent a similar amount of time in the light (Figure 5A), in the dark compartments (Figure 5B) and had a similar number of transitions from light to dark compartments (Figure 5C).

### TA1 Increases Mouse Spontaneous Locomotor Activity: Evidence for Histamine Involvement

The hole-board test is a validated model for evaluating the spontaneous locomotor activity and exploratory behavior of animals in a new environment (File, 1973).

**FIGURE 6:**
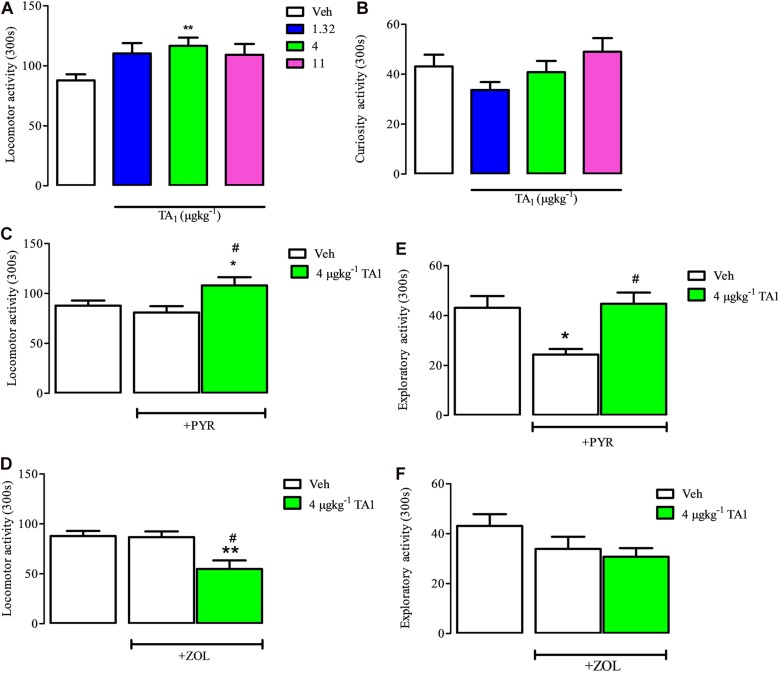
TA1 increases mouse spontaneous locomotor activity: evidence for histamine involvement. Mice were put on the hole board 15 min after 1.32, 4, and 11 μgkg^–1^ TA1 administration to evaluate the effect of the treatments on spontaneous locomotor activity and exploratory activities. Results are expressed as the means ± SEM of measurements relative to 10 mice. **(A,B)** The effect of TA1 treatment on spontaneous mice locomotion **(A)** and exploratory activity (curiosity, **B**) is showed (**P < 0.01 vs. One-way ANOVA test followed by Tukey Multiple Comparison Test). **(C,D)** Mice locomotion was evaluated in mice pre-treated i.p. with Veh or with pyrilamine (10 mgkg^–1;^ PYR; **C**) or with zolantidine (5 mgkg^–1^; ZOL; **D**),before receiving 4 μgkg^–1^ TA1 or Veh as described in Methods. Results are the means ± SEM of measures relative to 10 mice (*P < 0.05 and **P < 0.01 vs. Veh, #*P* < 0.05 vs. PYR/ZOL, One-was ANOVA test followed by Tukey Multiple Comparison Test). **(E,F)** Mice curiosity (exploratory activity) was evaluated in mice pre-treated with Veh or with PYR **(E)** or ZOL **(F)** before receiving 4 μgkg^–1^ TA1 or Veh. (*P < 0.05 and #*P* < 0.05 vs. PYR, One-way ANOVA test followed by Tukey Multiple Comparison Test).

Mice were put on the hole board platform 15 min after receiving 1.32, 4 and 11 μgkg^–1^ TA1 or Veh to measure the effect of the treatment on their locomotor and exploratory activities, i.e., curiosity (Figures 6A,B).

One-Way ANOVA analysis of the results indicated that only 4 μgkg^–1^ TA1 treatment, and not 1.32 or 11 μgkg^–1^, induced a significant increase of mice locomotor activity (**P < 0.01 vs. Veh treated mice). On the other hand, any of the TA1 doses administered modified the mice exploratory activity (curiosity) (Figure 6B).

Mice receiving PYR and ZOL showed locomotor activity similar to Veh-treated mice (Figures 6C,D, One-way Anova test followed by Tukey Multiple Comparison Test). We next verified whether TA1 stimulation of mice locomotion was modified in mice treated with PYR and ZOL.

In mice treated with PYR, 4 μgkg^–1^ TA1 conserved its capacity to stimulate mice locomotor activity (*P < 0.05 vs. Veh, #*p* < 0.05 vs. PYR, Tukey Multiple Comparison Test, Figure 6C), while in ZOL pre-treated mice, 4 μgkg^–1^ TA1 significantly reduced mice locomotor activity (**P < 0.01 vs. Veh, #*P* < 0.05 vs. ZOL; Tukey’s Multiple Comparison Test, Figure 6D).

PYR (10 mgkg^–1^), but not ZOL-treatment, induced *per se* reduction of mice exploratory activity (curiosity) (*P < 0.05 vs. Veh, Tukey’s Multiple Comparison Test; Figures 6E,F). Interestingly, in these mice, the administration of 4 μgkg^–1^ TA1 reverted PYR-induced depression of mice curiosity (Figure 6E; #*P* < 0.05 vs. PYR,). Instead, 4 μgkg^–1^ TA1 did not affect the curiosity of mice pre-treated with ZOL (Figure 6F).

### TA1 Does Not Produce Motor Incoordination

Mice receiving 4 and 11 μgkg^–1^ TA1 i.p. were then put on the accelerated rota-road as described in “Methods.” In these settings, mice treated with TA1 or with Veh showed similar performances with respect to the number of falls (data not shown), thus suggesting that TA1 did not compromise motor coordination.

## Discussion

We here demonstrate that TA1 shows brain bioavailability after systemic administration. The presence of TA1 in the brain is associated with the activation of the hypothalamic AKT and GSK-3β signaling pathways and the induction of emotional effects, including antidepressant-like effects. This result is independent on modulation of anxiety but it is associated with a mild stimulation of mice locomotion. Overall, these results demonstrate that TA1 is able to cross the BBB, a site where TA1 could activate mast cell degranulation. Indeed, TA1 systemic administration is associated with mast cell degranulation, and *in vitro* evidence demonstrates that TA1 may trigger histamine release from peritoneal mast cells. Interestingly, TA1 antidepressant-like effects and the stimulation of mice locomotion are modulated by anti-histaminergic drugs, thus confirming the dependence of these effects on the release of histamine. Even though the source of histamine involved in such effects (and in general with all the behavioral effects observed after administering TA1) remains elusive, our results provide further insight into the mechanism of TA1 action, suggesting that mast cells are a possible source of the histamine responsible for TA1 central-mediated behavioral effects.

TA1 is considered a by-product of thyroid hormone metabolism, produced by at least two independent synthetic pathways. However, little is known about the pattern of tissue distribution of endogenous and/or pharmacologically administered TA1. This issue has limited the exploration of the possible pathogenic or diagnostic role of this thyroid hormone metabolite in thyroid diseases. Our present results indicate that the endogenous levels of TA1 in mice brain and plasma are undetectable at our settings, whereas endogenous TA1 is highly detectable in liver. As expected, TA1 tissue levels increased following pharmacological administration. In particular, TA1 brain and plasma levels became detectable just 15 min after systemic administration, and, at this time point, a significant increase was also observed in liver as compared to control mice. These data provide evidence that TA1 is able to cross the BBB, and confirm that the liver is a preferential site not only of thyroid hormone but also of thyroid hormone metabolites accumulation (Porsolt et al., 1977; Saba et al., 2010). In line with this, our data also indicate that TA1 liver levels do not derive exclusively from local synthesis. Another interesting aspect of TA1 pharmacokinetic, is the different kinetic of tissue level reduction. Our data show that 60 min after administration, the decrease of TA1 brain levels was less than liver and plasma levels, suggesting that TA1 brain clearance might be lower than that of the liver. Moreover, even though the amount of TA1 recovered in the brain is only a small percentage of the dose administered, the brain may represent a site of “preservation of low levels” of TA1. These data are consistent with our previous observations (Laurino et al., 2015b) and suggest TA1 brain levels may be homeostatically controlled. However, the mechanism used by TA1 to cross the BBB and liver membranes remains to be investigated. Whichever mechanism is adopted, the passage of TA1 across the BBB implies an interaction with the cells lying on the brain side of the barrier, including mast cells, and the distribution in brain areas, including the hypothalamus where histaminergic neurons are highly concentrated. As a novel finding, our data demonstrate that TA1 has the capacity to degranulate mast cells and to trigger histamine release from peritoneal mast cells, showing a maximum effectiveness at 15 min after cell exposure and a minimum TA1 effective concentration of 20 nM. Notably, this concentration is in the range of the pharmacological doses of TA1 administered to mice, and the time of 15 min is also in line with the activation of the hypothalamic signals and the onset of the behavioral effects here described. Interestingly, the accumulation of histamine in cell medium did not depend linearly on the concentration of TA1. In fact, at 1 μM TA1, the histamine medium content did not increase further with respect to the amount released by 20 and 100 nM TA1, thus indicating a fast release of histamine in the absence of re-synthesis. The absence of a linear concentration-dependent effect might also explain the U-shaped dose-effect curves described for the *in vivo* effects of TA1 reported in literature. To note, mast cells express all the four subtypes of histamine receptors (Csaba et al., 2007) and can store thyroid hormone (Harvima et al., 2014). Overall, the fact that TA1, a thyroid hormone metabolite, may trigger histamine release from mast cells reinforces the hypothesis of a dual relationship between the endocrine, including the thyroid, and the immune system, a relationship which might have interesting clinical implications.

15 min after TA1 administration, an intracellular hypothalamic signaling is found to be activated. In particular, a 4 μgkg^–1^ TA1 dosage results in the activation of the AKT which is among the targets of mast cells-derived histamine (Kim et al., 2018), but it is also the cascade activated in the enhancement of cognition and the neuroprotection offered by type 3 histamine receptor antagonists/inverse agonists, whose effects are mainly due to the disinhibition of neuronal histamine release (Bitner et al., 2011; Bhowmik et al., 2014). At the same doses that are effective on signaling activation, TA1 induces antidepressant-like effects. As already observed in the case of pro-cognitive properties, these effects are prevented by pre-treating mice with an H1R antagonist (Lamberti et al., 1998). In addition, our results demonstrate that the antidepressant effect of TA1 is not a consequence of an anxiolytic effect and it is unrelated to serotonin, the amine co-stored with histamine in mast cells and strongly interplaying with neuronal histamine in the control of mood tone (Munari et al., 2015). Instead, we found that TA1 stimulates mice movements on the plane without giving motor incoordination. As for the antidepressant-like effects, mice locomotor activity results modulated by antihistaminergic drug treatment revealing a main role for the H2R activation. Interestingly, TA1 was also found to stimulate mice curiosity when the H1R is blocked, a condition which depresses, *per se*, mice curiosity. This finding highlights the role of TA1 in stimulating curiosity even when this is particularly depressed, a condition mimicking depression, and reinforces the involvement of the histaminergic system in TA1 behavioral effects. All the effects here described were observed in a the same narrow range of doses previously reported to stimulate memory (Laurino et al., 2015a; Bellusci et al., 2017), wakefulness (Laurino et al., 2018a), to protect neuron from excitotoxicity, to reduce PZT-induced seizures (Laurino et al., 2018b). Overall, these behavioral effects indicate that TA1 is a mild psychostimulant which, interestingly, rapidly increases the level of attention, the mood tone and cognition capacity of the subject without inducing motor incoordination.

In conclusion, our findings indicate that TA1 is a potent antidepressant-like drug displaying a rapid onset of action whose mechanism appears to involve brain histamine. Our data suggest that histamine may derive, at least in part, from brain mast cells. The observation that TA1 triggers histamine release from mast cells opens the way to the existence of a novel and not-yet-explored relationship between thyroid endocrine components, anxiety like behaviors and the immune system (Landucci et al., 2019). In addition, since mast cells are ubiquitous cells, the degranulating capacity of TA1 has to be taken into consideration when the pharmacological effects of TA1 are investigated.

## Data Availability

The raw data supporting the conclusions of this manuscript will be made available by the authors, without undue reservation, to any qualified researcher.

## Ethics Statement

Experiments and animal use procedures were in accordance with the National Institutes of Health Guide for the Care and Use of Laboratory Animals (NIH Publications No. 80-23, revised 1996). The experimental protocols were approved by the ethical Committee of the Italian Council of Health, in compliance with the European Convention for the Protection of Vertebrate Animals used for Experimental and Other Scientific Purposes (ETS no. 123) and the European Communities Council Directive of 24 November 1986 (86/609/EEC). The authors further attest that all efforts were made to minimize the number of animals used and their suffering.

## Author Contributions

LR designed the experimental protocol and wrote the manuscript. AL and EL performed the behavioral and biochemical experiments. LB performed the LC/MS-MS analysis. GC designed protocol for the analysis of TA1 in tissues. GD performed the drug administration and monitoring of the experimental settings. MG performed the Western Blot. LC performed the histochemistry experiments.

## Conflict of Interest Statement

The authors declare that the research was conducted in the absence of any commercial or financial relationships that could be construed as a potential conflict of interest.

## Abbreviations

BBB: blood brain barrier
FST: forced swim test
H1R: type 1 histamine receptor
H2R: type 2 histamine receptor
PCPA: p-chloro -phenylalanine
PYR: pyrilamine
T1AM: 3-iodothyronamine
TA1: 3-iodothyroacetic acid
TST: tail suspension test
ZOL: zolantidine.
